# Challenging Diagnosis of Streptococcus intermedius-Associated Empyema in an Immunocompetent Adult: A Case Report and Literature Review

**DOI:** 10.7759/cureus.60482

**Published:** 2024-05-17

**Authors:** Victor D Acuña-Rocha, Jenny C López-Zamarrón, Jose A Ramírez-Vázquez, Alejandro González-Castro, Erick J Rendón-Ramírez

**Affiliations:** 1 Internal Medicine, Hospital Universitario "Dr. José Eleuterio González", Monterrey, MEX; 2 Respiratory Medicine, Hospital Universitario "Dr. José Eleuterio González", Monterrey, MEX

**Keywords:** pleural drainage, immunocompetent host, pleural effusion, empyema, streptococcus intermedius

## Abstract

The significance of *Streptococcus intermedius* in infectious diseases, especially pleural infections, is gaining recognition. While traditional risk factors like dental procedures and immunosuppression remain pivotal in differential diagnosis, there is an emerging recognition of unconventional clinical presentations and risk factors linked to infections by *S. intermedius*. This shift compels medical professionals to broaden their diagnostic and therapeutic strategies, underscoring the intricate and evolving nature of managing infections associated with this opportunistic bacterium.

We describe the case of a 48-year-old immunocompetent woman with untreated hypertension who experienced a 15-day episode of right-sided chest pain, which worsened with a sudden onset of dyspnea, yet her daily activities remained unaffected. Physical examination suggested a pleuropulmonary syndrome due to significant pleural effusion, with a computed tomography (CT) scan of the lungs revealing about 50% effusion on the right side. Laboratory tests indicated elevated inflammatory markers. Ultrasound-guided thoracentesis extracted purulent fluid compatible with empyema, necessitating the placement of a pleural drain and multiple pleural cavity lavages using alteplase, which led to the removal of substantial infected fluid. Culture of the pleural fluid identified *S. intermedius*, which was pansusceptible. Treatment with intravenous ceftriaxone was administered, resulting in a favorable clinical outcome. This case highlights the critical nature of recognizing atypical clinical presentations and managing complex bacterial infections in the pleural space.

## Introduction

The role of Streptococcus intermedius in pleural infections is increasingly recognized [1]. Traditional risk factors such as dental procedures and immunosuppression remain critical for the differential diagnosis when this bacterium is involved [2,3]. Additionally, there is growing awareness of atypical clinical presentations and non-traditional risk factors associated with S. intermedius infections [4]. This case is particularly unique as it presents a rare instance of pleural infection caused by S. intermedius without pulmonary parenchymal involvement in an immunocompetent patient, highlighting the need for heightened diagnostic vigilance.

## Case presentation

We present the case of a 48-year-old immunocompetent woman with a history of untreated systemic arterial hypertension, who began experiencing symptoms in April 2024. She had a 15-day history of right-sided chest pain, which she rated as 7 out of 10 on the Visual Analog Scale (VAS). The pain did not limit her activities and remained constant in intensity, unaffected by physical activity, effort, or time of day. However, it worsened five days prior to her initial evaluation, following the insidious onset of grade 2 dyspnea on the mMRC scale, prompting her to visit the emergency department. Upon arrival, her vital signs were as follows: a blood pressure of 140/90 mmHg, a heart rate of 89 beats per minute, a respiratory rate of 19 breaths per minute, a body temperature of 37.5°C, and an oxygen saturation of 96% on room air.

**Figure 1:**
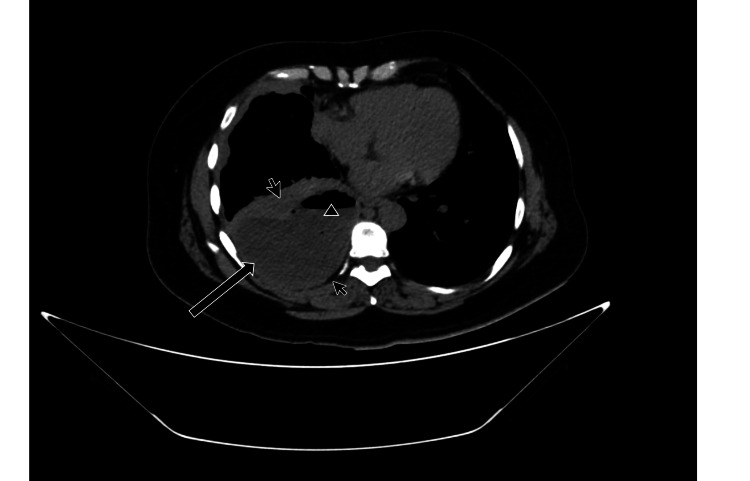
CT pulmonary axial view. Black arrow: Heterogeneous hypodense material in the pleural space. Small arrows: Pleural thickening associated with subpleural fat space preservation. Arrowhead: Hydro-air level.

**Figure 2:**
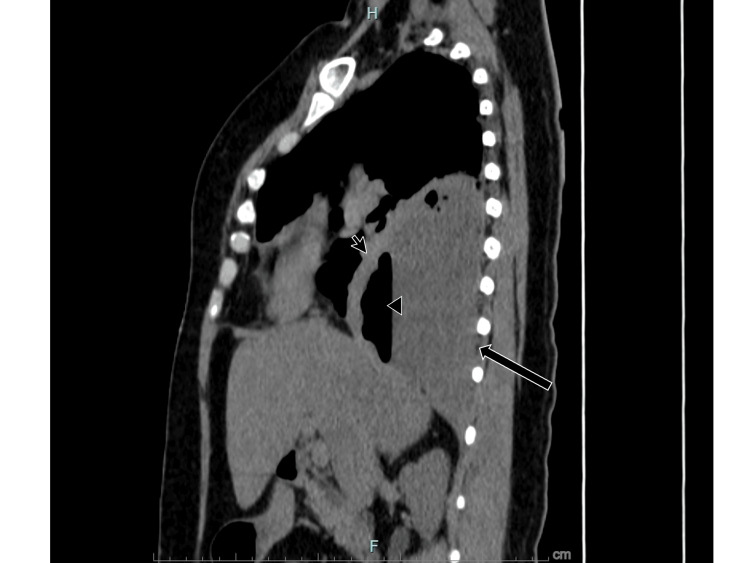
CT pulmonary sagittal view. Black arrow: Heterogeneous hypodense material in the pleural space. Small arrow: Pleural thickening. Arrowhead: Hydro-air level.

Physical examination revealed pleuropulmonary syndrome consistent with pleural effusion. Subsequently, a pulmonary computed tomography (CT) scan was performed. A heterogeneous hypodense area with varied densities was observed in the axial images of the right hemithorax, alongside pleural thickening, air bubbles, and hydro-air levels. Moreover, the subpleural fat preservation sign was noted, suggesting empyema (Figures 1, 2).

We conducted general laboratory tests upon admission, as detailed in Table 1. Additionally, we performed ultrasound-guided thoracentesis, yielding 100 ml of thick purulent fluid. Analysis of this fluid revealed a white cell count of 1,344,000 cells/mcL, with 85% polymorphonuclear neutrophils. Glucose was measured at 8.0 mg/dL, protein at 2,100 mg/dL, and lactate dehydrogenase at a significantly elevated level of 9,927 IU/L. Gram staining identified Gram-positive cocci. Furthermore, the Ziehl-Neelsen stain tested negative for acid-fast bacilli, and cultures showed no growth of mycobacteria.

**Table 1:** Initial laboratory results for the patient at admission.

Category	Test	Result	Normal Range
Blood Counts	Hemoglobin (mg/dL)	15	12.1-15.1
	Hematocrit (%)	47.6	36.1-44.3
	White Blood Count (K/mcL)	12.9	4.5-11.0
	Neutrophils (K/mcL)	11.5	1.8-7.8
	Platelets (K/mcL)	348	150-450
Metabolic Panel	Creatinine	0.9	0.59-1.04
	Blood Urea Nitrogen (mg/dL)	35	7-20
	Glucose (mg/dL)	260	74-106
Inflammatory Markers	Erythrocyte Sedimentation Rate (mm/hr)	32	0-29
	C-Reactive Protein (mg/dL)	9.9	<1.0
Protein Levels	Albumin (g/dL)	3	3.4-5.4
Blood Gases	pH	7.44	7.35-7.45
	pO_2_ (mmHg)	79	75-100
	pCO_2_ (mmHg)	36	35-45
	Bicarbonate (mmol/L)	24.5	23-30

**Figure 3:**
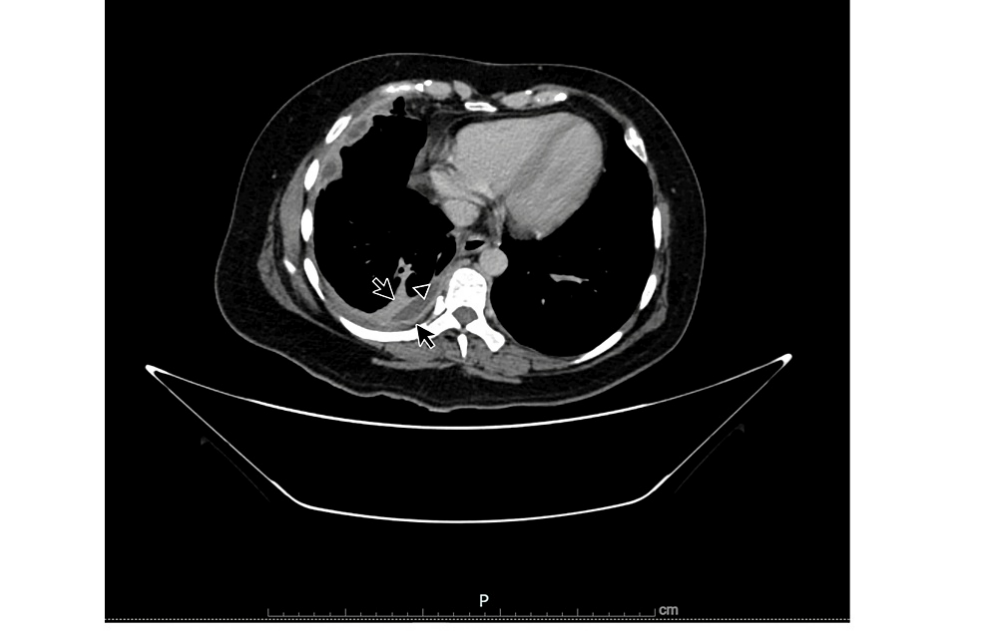
CT pulmonary axial view. Arrowhead: Heterogeneous hypodense material in the pleural space. Small arrows: Pleural thickening along with split sign.

**Figure 4:**
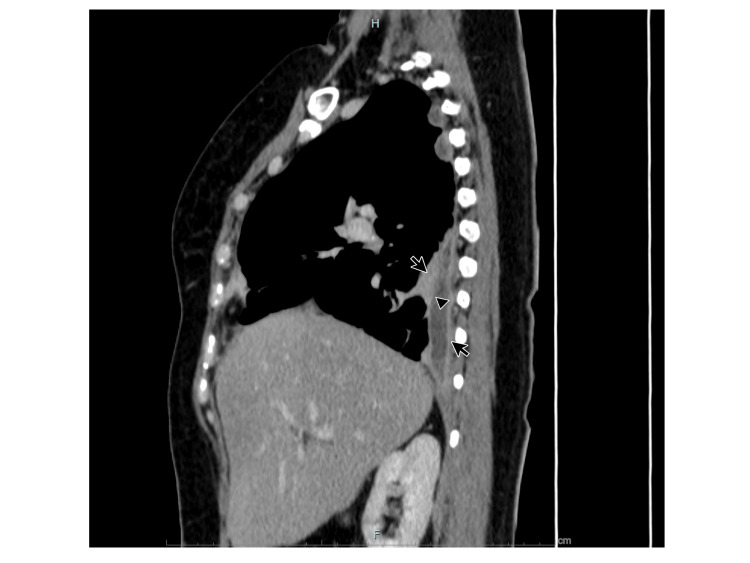
CT pulmonary sagittal view. Arrowhead: Heterogeneous hypodense material in the pleural space. Small arrows: Pleural thickening along with split sign.

The condition was identified as empyema, requiring the placement of a single catheter pleural drain. The patient was treated with intrapleural alteplase, resulting in pleural cavity lavage on five separate occasions and the drainage of 1,500 ml of fluid. A follow-up pulmonary CT contrast scan performed six days after the initial treatment showed a 90% reduction in the pleural effusion, and a hyperenhancing thickening of the visceral and parietal layers of the pleura, separated by a collection of fluid, was noted, consistent with the split pleura sign (Figures 3, 4).

Pleural fluid culture isolated S. intermedius with susceptibility to ceftriaxone, cefotaxime, and vancomycin, although minimum inhibitory concentration (MIC) values were not reported. Given the microbial isolation, the patient was managed with intravenous ceftriaxone 2 g every 24 hours for 15 days, showing appropriate clinical response to the medical treatment. Consequently, she was discharged. Two weeks post-discharge, during a follow-up, the patient reported a pleuritic pain rated 2 out of 10 on the VAS. Pulmonary ultrasound was performed, showing complete resolution of the pleural effusion. Four weeks after her discharge, the pleuritic pain had completely resolved. Currently, the patient remains asymptomatic.

## Discussion

Infectious pleural effusions are predominantly associated with Staphylococcus aureus, followed by Streptococcus viridans, Pseudomonas, Enterobacteria, and Streptococcus pneumoniae [5]. Regarding S. intermedius, which is part of the Streptococcus anginosus group (SAG), its prevalence is about 7.7% in a retrospective study [6].

The SAG, which includes S. anginosus, S. constellatus, and S. intermedius, is typically found in the oral cavity and can cause serious infections when they translocate to sterile body sites. Historically considered mere commensals, recent insights have revealed their potential as opportunistic pathogens capable of causing severe infections such as bacteremia, abscesses, and empyema [3]. Despite the diversity among the organisms in this group, members of the SAG share some common microbiological characteristics: (i) slow growth in culture media, (ii) a characteristic "caramel" scent due to arginine hydrolysis, and (iii) production of acetoin from glucose [2].

Several virulence factors have been described for S. intermedius, including the ability to develop biofilms for protection against microbial agents and the immune system; production of hydrolytic enzymes such as hyaluronidase and chondroitin sulfate depolymerase, capable of degrading glycosaminoglycans; and glycosidases such as N-acetylneuraminidase and β-D-galactosidase, which enable growth on macromolecules found in the reservoir [7].

Regarding the types of infections linked to the isolation of S. intermedius, they are primarily purulent, predominantly involving the head, neck, and pleuropulmonary areas. These often extend to the lung parenchyma manifesting as pneumonia and are associated with empyema [1-4,8]. However, there are limited reports of cases presenting solely with isolated infectious pleural effusion [4], as observed in our patient. This highlights the importance of clinical suspicion for this microorganism with only the clinical presentation of pleural effusion.

In diagnosing infections caused by S. intermedius, differentiating this pathogen from other members of the SAG poses a considerable challenge. Initial diagnostic steps include standard phenotypic methods like hemolytic activity on blood agar, yet these are inadequate for differentiation within the SAG. Consequently, species-specific PCR that targets genes such as ily, which is unique to S. intermedius, is considered the gold standard. Moreover, advanced techniques like matrix-assisted laser desorption ionization-time-of-flight (MALDI-TOF) mass spectrometry also offer rapid and precise species-level identification, as demonstrated in our patient who was identified through sequencing and MALDI-TOF [9].

For infections suspected of involving deep tissues or the central nervous system, imaging techniques like CT or magnetic resonance imaging are crucial to localize infections and assist in the management planning process [2].

Additionally, most patients have predisposing factors such as immunosuppression, advanced age, male sex, alcoholism, intravenous drug use, and dental manipulation, as described by Dyrhovden et al. in 2023 in an analysis of 43 patients with pleural infection by S. intermedius [1], and as described by Cobo et al. [4], unlike our patient who had none of these. This is imperative when considering the diagnostic approach to isolated empyema in immunocompetent patients without the usual risk factors associated with this bacterium.

The management of S. intermedius pleural infections typically involves a combination of surgical and antimicrobial strategies. The cornerstone of treatment is the use of appropriate antimicrobial therapy guided by susceptibility testing, with common regimens including agents like penicillin, amoxicillin-clavulanate, ceftriaxone, metronidazole, and occasionally vancomycin, depending on the resistance profile [2,3].

In association with antimicrobial treatment, for patients with complicated pleural effusion such as our patient presenting with empyema, it is recommended to combine drainage using small-caliber endopleural tubes (<14 Fr) as part of interdisciplinary management [10,11] and the administration of alteplase and DNase as part of the aforementioned treatment [12].

Despite the rarity of this pathogen associated with an atypical presentation of this disease, the patient had a favorable outcome as she was a candidate for placement of a small-caliber pleural catheter and performed cavity lavages on five occasions with the administration of intrapleural alteplase, achieving total drainage of approximately 1,500 ml and showing significant improvement in control imaging studies alongside her clinical condition. She continued to progress well with no relapses in her outpatient follow-up visits.

Regarding the strengths, this case report effectively delineates the characteristics and virulence factors of S. intermedius, particularly emphasizing its role within the SAG. The comprehensive microbial profiling provided is instrumental in understanding how this typically commensal bacterium transforms into an opportunistic pathogen capable of causing severe infections. Furthermore, the incorporation of advanced diagnostic techniques, such as PCR and MALDI-TOF mass spectrometry, is also highlighted as a significant strength. These techniques are critical for the precise identification of the pathogen, especially valuable given the challenges in differentiating members of the SAG, thus enhancing diagnostic accuracy. However, the report also acknowledges some limitations, such as the lack of complete microbiological data, specifically MIC values, which are crucial for a thorough understanding of antibiotic resistance.

## Conclusions

Pleural infections without involvement of the lung parenchyma require a deeper understanding of less common etiologies. Even without traditional predisposing factors such as immunosuppression, dental procedures, or intravenous drug use, this patient's development of empyema highlights the opportunistic nature of S. intermedius and its ability to cause serious infections in immunocompetent individuals. Our discussion underscores the complexity of diagnosing infectious pleural effusions, particularly those with atypical presentations, and the importance of considering a broad differential to ensure earlier and more effective treatment.
